# Distance estimation in the goldfish (*Carassius auratus*)

**DOI:** 10.1098/rspb.2022.1220

**Published:** 2022-10-12

**Authors:** Adelaide Sibeaux, Cecilia Karlsson, Cait Newport, Theresa Burt de Perera

**Affiliations:** Department of Biology, University of Oxford, Zoology Research and Administration Building, 11a Mansfield Road, Oxford, Oxfordshire OX1 3SZ, UK

**Keywords:** distance estimation, teleost, optic flow, navigation, vision

## Abstract

Neurophysiological advances have given us exciting insights into the systems responsible for spatial mapping in mammals. However, we are still lacking information on the evolution of these systems and whether the underlying mechanisms identified are universal across phyla, or specific to the species studied. Here we address these questions by exploring whether a species that is evolutionarily distant from mammals can perform a task central to mammalian spatial mapping–distance estimation. We developed a behavioural paradigm allowing us to test whether goldfish (*Carassius auratus*) can estimate distance and explored the behavioural mechanisms that underpin this ability. Fish were trained to swim a set distance within a narrow tank covered with a striped pattern. After changing the background pattern, we found that goldfish use the spatial frequency of their visual environment to estimate distance, doubling the spatial frequency of the background pattern resulted in a large overestimation of the swimming distance. We present robust evidence that goldfish can accurately estimate distance and show that they use local optic flow to do so. These results provide a compelling basis to use goldfish as a model system to interrogate the evolution of the mechanisms that underpin spatial cognition, from brain to behaviour.

## 1. Introduction

Key neural structures that underpin navigation have been discovered in mammals, birds and reptiles (reviewed in [1]). The ground-breaking discovery of place and grid cells in the late twentieth century changed the way we thought about the encoding of space [2–4]. These neural cells, located in the mammalian hippocampal formation, allow an individual to obtain information about its position in space by creating an internal map of its environment. Similar neural circuits have been found in birds [5] and reptiles [6] suggesting the existence of a common ground plan of a ‘hippocampal formation-like circuit’ in ancestral amniotes [1]. Teleost fish are comprised nearly 30 000 species [7] and are the most successful and diverse group of vertebrates, inhabiting various ecological niches and presenting a high level of morphological, physiological and behavioural diversity. However, the neurological structures and functions linked to spatial cognition in teleosts have only been investigated recently (see [8]), and the neural basis of spatial cognition in teleost fish is still far from understood. This crucial information would allow us to build a more cohesive picture of the evolutionary origin of spatial navigation and its underpinning cells.

Neuroanatomical studies have shown that lesions in the lateral pallium affect the navigational performance of goldfish (*Carassius auratus*) [9,10]. Similar results were found when the hippocampal formation of rats was lesioned [10], indicating that these structures are homologous. Neural cells that are likely to constitute the basic building blocks of fish navigation systems were recently discovered in the goldfish lateral pallium; head direction cells, edge detection neurons and speed correlated cells were recorded when individuals freely navigate in their environment [11]. Crucially, we still do not know whether grid and place cells, the two main cell types used in mammalian spatial mapping and navigation, are present in teleost fish.

An essential step towards addressing this gap is to understand the spatial behaviour of teleost fish, the final output of spatial processing within the brain. Determining whether teleosts can navigate efficiently by computing moving distance and direction information, and the sensory mechanisms associated with these behaviours will inform us about the neural structures that are likely to underpin them. Here, we use a behavioural paradigm to test whether the spatial metric of distance is encoded by a species of fish (goldfish: *Carassius auratus*), with the intention of developing a new model system that can be used to link together behaviour with the neural mechanisms that drive it. Goldfish have already been used as a neural model for spatial cognition [8,9,11–13]. They are easily accessible, and they are capable of learning, showing spatial, social and numerical cognitive abilities [14]. This study aims to first determine whether goldfish are able to estimate distance travelled, making them a prime model system to explore space mapping in teleost fish and second, to test the sensory mechanisms behind this ability.

To estimate distance travelled, terrestrial species can use various, non-mutually exclusive, behavioural mechanisms such as stride integration [15–17], internal vestibular movements [18,19], energy use [20] or self-induced optic flow [21–24]. The latter mechanism refers to the degree of visual change due to the motion of an individual through its environment. It can be measured by animals in two ways: either by measuring the angular speed of visual features across the retina, referred to as ‘global optic flow’ or by measuring the spatio-temporal frequency of visual features in the environments (i.e. visual density), referred to as ‘local optic flow’. Global optic flow depends on the individual speed and its distance from the visual background but is independent of variation in the environmental spatial frequency. Local optic flow is widely used to control the optomotor and optokinetic responses [25] (for more details see [26]). Previous results have shown that Picasso triggerfish (*Rhinecanthus aculeatus*) are able to accurately estimate travelled distance using local optic flow as an odometer [27,28]. Crucially, however, it is unknown whether goldfish, the potential model species to link neural and behavioural understanding of teleost navigation, possess the same abilities. It is possible that distance estimation is phylogenetically conserved across fish species. However, it is also possible that the environment might drive the evolution of the spatial mapping system. Goldfish and Picasso triggerfish inhabit very different environments and differ in many major behavioural traits. While the social goldfish inhabit water ponds with reduced colour saturation, the territorial triggerfish are found in clear, bright and highly colourful reef water. The difference in how they might use space, and the visibility of navigational cues could lead to major divergence in their spatial mapping capabilities and underlying mechanism. We tested (i) whether goldfish are able to accurately estimate travel distance, confirming their utility as a robust model species to investigate the neurophysiological basis of distance estimation in teleost fish; (ii) whether they use visual motion information (global or local optic flow) to estimate distance, which we suggest as they are a highly visual species [29] and (iii) whether alternative mechanisms, including travel time and number of fin beats, are used for distance estimation.

## 2. Material and methods

### (a) Experimental overview

We trained goldfish to reach a target distance in a long and narrow tank and then tested whether they could continue to swim to the target distance following manipulation of the visual background. In training, the fish were exposed to an achromatic vertical striped pattern of 2 cm, and they were given an external cue to indicate when they reached the target distance. We then removed the external cue and measured if the fish continued to swim the set distance. Finally, we changed the visual background pattern to determine whether the fish would change their estimate of distance travelled. Trial videos were then analysed to test whether alternative mechanisms, including fin beats and time, could have been used for distance estimation.

### (b) Animal husbandry

Nine naive goldfish (*Carassius auratus*), sourced from a local supplier (The Goldfish Bowl, 118–122 Magdalen Road, Cowley, Oxford OX4 1RQ, UK), were used in experiments. Individuals were reared in wide indoor ponds enriched with natural plants by the supplier. In the laboratory, individuals were housed in 0.35 m × 0.32 m × 0.60 m (width × height × length) tanks enriched with 0.5 cm of gravel, a terracotta pot and plastic plants. Because *C. auratus* is a social species, individuals were kept in groups of two to three fish. The illumination by fluorescent light followed a 12 h light/12 h dark cycle. Individuals were fed twice a day; once in the morning with pellets (*Fancy Goldfish Sinking Pellets, FishScience*) and once in the afternoon with spinach or bloodworms to add supplementary nutrients. Tanks were cleaned weekly and water quality was maintained at healthy levels for this species (pH: 8.2; KH: 7dKH; GH: 8.2; Nitrite: 0 ppm; Ammonia, Nitrate: less than 10 ppm).

### (c) Experimental apparatus

**Figure 1.**
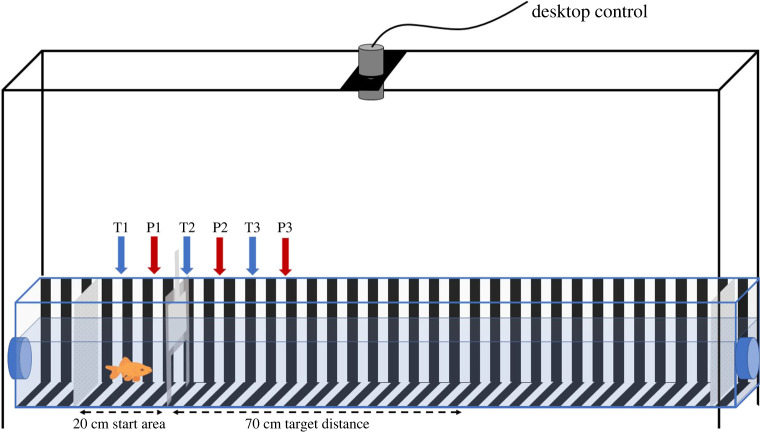
Experimental set-up to test distance estimation in the goldfish. Black and white striped panels (width 2 cm) covered the tank walls and floor providing constant optic flow information to the individual (only floor and left-side wall are represented for clarity). The fish was placed in a movable start area, with a sliding door, for acclimation. Once the door opened, the fish was trained to swim until the experimenter waved above the tank at the 70 cm target distance and then to return to the start area to receive a food reward. Two white partitions (grey on picture for clarity) prevented the fish to obtain external visual cues from both ends of the tunnel, one was placed at the end of the experimental tank and the other one was placed 20 cm behind the starting door. An overhead camera (grey cylinder) connected to the laboratory computer displayed fish movement in real time and recorded distance estimates during the testing phase. The linear tunnel was constructed inside a flow-through tank, with water flow in behind the start area (left blue pipe) and passive water flows out at the opposite end of the tank (right blue pipe). Water flowed outside of training and testing sessions. T1, T2 and T3 indicate the three start positions used during training. P1, P2 and P3 indicate the three start positions used in test. (Online version in colour.)

We used the experimental apparatus built by Karlsson *et al*. [27]. Briefly, fish were trained and tested in an acrylic tank (0.25 m high × 0.16 m wide × 1.80 m length; figure 1) set within a flow-through tank. The tank was connected to the home water system to maintain consistent water parameters, but the water flow was stopped during training and testing sessions. A white and black vertical 2 cm width stripe pattern (2 cm pattern) on the floor and walls of the tunnel provided optic flow cues. Three additional patterns altering either the geometry or the spatial frequency of the background pattern were used to interrogate which visual features were used by the goldfish. (i) *Checker pattern*: a 2 cm^2^ checkerboard pattern with the same spatial frequency as the training pattern but a different geometry was used to evaluate the impact of pattern change on fish distance estimation. (ii) *High spatial frequency pattern*: a 1 cm width vertical stripe pattern, used to test whether altering the spatial frequency of the visual background affected distance estimation. (iii) *No periodic spatial modulation pattern*: a 2 cm horizontal stripe pattern used to test the impact of removing translational optic flow information on distance estimation.

To control for the use of external visual cues to estimate distance, a movable start area (0.25 m high × 0.16 m wide × 0.20 m length) was placed at one of six start positions (10 cm apart). Three start positions (20 cm apart) were used to train the fish and three different start positions (20 cm apart) were used to test them. Therefore, while the target travel distance remained constant, the absolute position where the fish must turn was different in training and testing. A white partition was placed at the end of the tunnel to block external visual stimuli, and a second one was placed 20 cm behind the start door. An overhead camera (Point Grey GrassHopper 3M- FLIR Machine Vision Cameras), placed 1.05 m above the water level and connected to a computer displaying individual movement in real time. All training and testing trials were recorded using StreamPix 7 video capture software (video frame rate = 50 fps).

### (d) Training

We used an operant training paradigm with a food reward to train the fish to swim to a target distance of 70 cm. This distance is significantly different from linear travel bouts travelled by naïve goldfish (mean ± s.d. = 24.78 ± 14.89 cm, see electronic supplementary material §1 for details). For each training session, the start area was randomly assigned to one of the three training start positions. During the first stage of training, bloodworms were placed along the bottom of the tank to encourage the fish to swim through. A transparent acrylic barrier was placed 70 cm apart from the door to stop the fish swimming further than the target distance. Bloodworms were then spaced throughout the tunnel to provide motivation for exploration. The number of bloodworms gradually decreased throughout training until only one blood worm was placed at the barrier and one at the start position. Gradually, the perplex barrier was replaced by a 5 cm partition, then a 0.5 cm stick, and finally the physical barrier was removed from the tank altogether. Those intermediate steps were necessary as they provided a physical cue indicating that the target distance was reached but also allowed the fish to get around it, swim further than the target distance and explore the tank. If the fish swam further than the target distance, no food reward was provided when it came back to the start position. The experimenter stood 1 m apart from the experimental tank, observing the fish movements on the computer screen and was not visible to the fish. The experimenter waved at the fish when it reached the target distance (with the 5 cm partition, 0.5 cm stick and without a physical barrier) to provide a cue for turning. At the final stage of training, the fish had to swim and turn when the experimenter waved above the experimental tank and then come back directly to the start position to receive their food reward. If the fish returned to the start position before reaching the target distance or explored the tunnel further away, no food reward was given. The training was completed once a fish reached the target distance in 80% of trials (i.e. swim out to the wave and come back directly to the start area) over three consecutive sessions. Training sessions lasted for 10 min or until 10 trials were completed. Fish were trained twice a day, once in the morning and once in the afternoon, 5 days a week.

### (e) Testing

During testing, three start positions (different from the three training start positions) were randomly assigned and 15 trials per fish were completed at each start position. As a result, 45 distance estimation tests were recorded for each fish. For each session, before testing the fish first performed six to seven training trials with the experimenter providing a turning cue. The training start position was then moved to the test start position and the fish were tested for three to four trials. Turning cues were not available during distance estimation test trials, which were rewarded regardless of the distance travelled. To test the importance of optic flow in distance estimation and to determine the visual features used by the fish, individuals were returned to their home tank after training while the experimenter changed the background pattern. Fish were then moved back into the experimental tank and tested for a further three to four trials. A total of 45 distance estimation tests were also recorded for each background pattern.

A trial was discarded (i) if the fish reached the very end of the experimental tank (5.50%) or if it turned in less than a body length of the start position (7.45%). (ii) If the fish turned multiple times in the tunnel before returning to the start position (9.35%), or (iii) if the fish showed erratic swimming movement indicating stress for the individual (0.95%). In this final case, the trial was discarded, the session was ended and the fish returned to its home tank and monitored. Six of the nine fish were tested with four different patterns. The power achieved to test the effect of the change of background pattern on distance travelled with six individuals was above the 80% threshold criteria (power for 1000 iterations of the generalized linear mixed model = 94,50%, confident interval_95%_ [92.90; 95.83], R package *simr*, [30]).

### (f) Data collection

The videos were recorded using StreamPix7 software. To measure the goldfish distance estimate for each test trial, frames were extracted from video recordings at the point when the fish exited the start area and when it turned in the experimental tank. The pixel coordinates of the fish mouth were then manually recorded for those two events using custom video tracking software (Matlab version R2022a, MathWorks Inc.). The difference in pixel coordinate between the exit of the start area and the turn position was then converted into a distance estimate measured in centimetres (ratio 1 pixel = 0.0664 cm). The absolute turn position was measured using the coordinate of the turn frame converted in centimetres.

The time taken to turn in seconds was measured using the number of frames elapsed between the exit of the start area and the turn position. The frame rate (50 fps) was used to convert the number of frames to seconds. The number of caudal fin beats for each test trial was manually counted by the experimenter using StreamPix7 software that allowed frame-by-frame video inspection.

To determine if the goldfish used optic flow cues to estimate distance, we compared the average turning distance and the absolute turn position obtained with the 2 cm pattern, the checker pattern (providing same spatial frequency), the 1 cm vertical stripes pattern (high spatial frequency) and the horizontal stripes pattern (no periodic spatial modulation).

### (g) Statistical analyses

All statistical analyses were performed in *R* (v.4.0.2, 2020) with *R Studio* (R Studio 2009–2020, v.1.3.1056). Normality and homogeneity of the residuals were successfully verified for each linear mixed model before further analysis. For each model detailed below (GLMM and LMM), we tested three different model structures with either ‘individual’ as a random intercept, ‘individual’ and ‘start position’ as crossed random intercepts or ‘individual’ as a random intercept and ‘start position’ as a random slope. We compared models using the AIC criteria and performed analyses of variance between each model pair [31] to select the best model structure. All model AIC values are presented in the electronic supplementary material, §2. We also ran a *post hoc* analysis on the selected model to control for multiple comparisons using the glht function and Holm-Bonferroni adjustment (R package *multcomp*, [32]). A power test (R package *simr*, [30]) was performed *post hoc* to verify that the number of individuals tested was sufficient to produce enough statistical power for following each changed in background pattern. All tests were conducted with alpha = 0.05. We used the package *ggplot2* to draw figures [33].

#### (i) Overall distance estimation accuracy

To evaluate the goldfish distance estimation accuracy, individual average distance travelled and standard deviation was measured. We also performed a one-sample *t*-test on the 9 average values against the target distance mu = 70. Normality of the data was preliminarily verified using a Shapiro–Wilk test (*W* = 0.97, *p* = 0.915).

As a control, we tested if the order of the trial test had an effect on the distance travelled. A linear mixed-effects model (R package lme4 [34]) was performed with distance travelled included as the response variable and test order as the fixed effect. The model structure that fitted our data best included ‘individuals’ and as random intercept and ‘start position’ as a random slope.

#### (ii) Robustness of distance estimation across start position

To evaluate if the fish turned at the estimated target distance or if it learnt to turn using a landmark cue outside or inside the experimental tank, we tested if the start position affected the absolute turning distance. We performed a linear mixed-effects model (lmer package lme4 [34]) with absolute turning distance as the response variable and start position as the fixed effect. The model structure that fitted our data the best included ‘start position’ and ‘individuals’ as a random slope and intercept, respectively.

#### (iii) Alternative cues used for distance estimation

To test the effect of three alternative cues: start position, number of fin beat and travel time on distance travelled, we performed a linear mixed model (lmer package lme4 [34]) with distance travelled as the response variable and start position, fin beats number and time as the fixed effects. The model structure that fitted our data the best included ‘individuals’ and as a random intercept.

Because travel time and fin beat number are likely to increase with travelled distance, we measured the relative variation of distance travelled and either travel time or fin beat number. We measured the coefficient of variation (standard deviation/average × 100) of distance travelled versus travel time/fin beats, and then determined the ratios.

As a control, we tested if the order of the test trial had an effect on the travel time. We performed a generalized linear mixed model (R package lme4 [34]) with a Gamma family to account for the elongated tail structure of the travel time data. Travelled time was included as the response variable and test order as the fixed effect. The model structure that fitted our data the best included ‘start position’ and ‘individuals’ and as a random slope and intercept, respectively.

#### (iv) The use of background visual information

To evaluate if goldfish used optic flow information to estimate distance, we performed a linear mixed model (R package lme4 [34]) with distance travelled as the response variable and background pattern as the fixed effect. The model structure that fitted our data the best included ‘start position’ and ‘individuals’ and crossed random intercept.

We ran a linear mixed model (R package lme4 [34]) to evaluate if the swimming speed (distance travelled/time) was affected by the background pattern. Speed was added in the model as the response variable and background pattern as the fixed effect. The model structure that best fit our data included ‘individuals’ as a random intercept.

## 3. Results

### (a) Overall distance estimation accuracy

**Figure 2.**
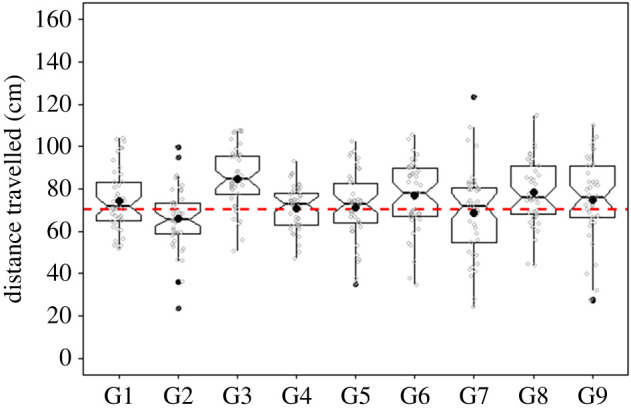
Distance estimated for the nine goldfish tested with the 2 cm pattern. Black dots represent the mean distance estimated for each fish. The dashed line at 70 cm represents the target distance. Raw data are represented by the grey dots. *n* = 45 test trials per fish. See electronic supplementary material, table A1 for details. Histograms of the distance estimate are presented in the electronic supplementary material, figure A1. (Online version in colour.)

All nine goldfish were able to reach the training criteria within three–five months (approximately 120 sessions and 1200 trials per fish) and complete testing. During testing, the same pattern as in training was used (2 cm vertical stripes). The fish turned back towards the start position once it estimated that it had swam the target distance. No external experimenter cues were provided at this stage. On average, the goldish swam 73.99 ± 16.77 cm (mean ± s.d., target distance = 70 cm, *n* = 405 trials) before turning (figure 2; electronic supplementary material, table A1 and figure A1). Only 1 individual (fish ID: G3), swam an average of 84.75 ± 13.92 cm and did not show an overlap of the target distance of 70 cm. The grouped mean distance travelled was not significantly different from that of the target distance (one-sample *t*-test, *t* = 2.12, d.f. = 8, *p* = 0.066, CI_95%_ [69.65; 78.32]). We did not find any effect of the test order on distance travelled (*p* = 0.708, see electronic supplementary material, table A2 for details).

### (b) Distance estimation is robust to changes in start position

**Figure 3.**
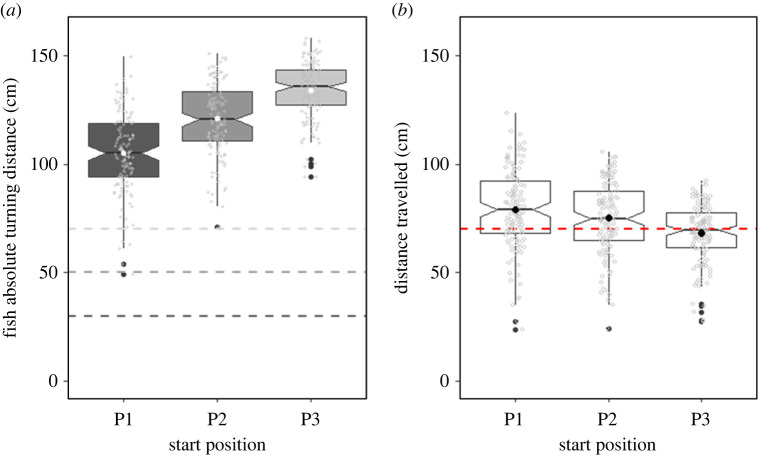
Goldfish distance estimation accuracy as a function of start distance with the 2 cm pattern. Raw data are represented by the grey dots. (*a*) Absolute turning distance at three start positions. The dashed lines represent the three start positions. White dots represent the mean absolute turning distance for each start position. See electronic supplementary material, figure A2 for individual details. (*b*) Distance travelled per start position. The black dots represent the mean distance travelled. The dashed line represents the 70 cm target distance. See electronic supplementary material, figure A3 for individual details. (Online version in colour.)

The start positions significantly affected the individual absolute turn distance (turning point in the experimental tank) (figure 3*a*; table 1). Consistent with the fish accurately estimating distance, the further the fish started within the tank, the further they turned. Absolute swimming distances were 104.86 ± 18.25 cm, 120.93 ± 16.55 cm and 134.06 ± 13.40 cm when the fish started at the first, second and third position respectively.

**Table 1.** Effect of the start position on the absolute turn position in the experimental tank. Adjusted *p*-values (Holm method) are reported. Results from the linear mixed model with fish ID as random intercept and position as random slope. Significant results are in italics. d.f. = degrees of freedom; *p* = *p-*value; s.d. = standard deviation; s.e. = standard error; *t* = *t* value. 95% confidence intervals of the bootstrapped medians (resampling *n* = 1000) : P1 CI_95%_ [100.46; 108.37], P2 CI_95%_ [118.23; 125.66], P3 CI_95%_ [132.77; 138.26].

fixed effects	*β* coefficient	s.e.	d.f.	*t*	*p*
intercept	104.86	2.87	8.00	36.59	*<0.001*
position (P2–P1)	16.07	2.75	8.07	5.85	*<0.001*
position (P3–P1)	29.20	3.07	8.09	9.53	*<0.001*
position (P3–P2)	13.13	2.53	9.94	5.19	*<0.001*
random effects	variance	s.d.	R		
individual (intercept)	58.85	7.67			
position (P2–P1)	37.77	6.15	−0.43		
position (P3–P1)	54.40	7.38	−0.94		
position (P3–P2)	27.47	5.24	−0.98		
residuals	225.95	15.03			

### (c) Alternative cues used for distance estimation

We tested the effect of start position, fin beats number and time on goldfish distance travelled. Fish swam a significantly shorter distance when they started from the third position (68.06 ± 13.43 cm) than from the first (78.82 ± 18.24 cm) or second (75.07 ± 16.56) position (table 2; figure 3*b*). There was no significant difference between the first and second start position. Individual were closer to the target distance and more accurate in their distance estimate at the third position. Individual distances travelled at each start position are presented in the electronic supplementary material, figure A3.

**Table 2.** Effect of travel time, number of fin beats and start position on goldfish distance travelled. Adjusted *p*-values (Holm method) are reported. Results from the linear mixed model with fish ID as random intercept. Significant results are in italics. d.f. = degrees of freedom; *p* = *p-*value; s.d. = standard deviation; s.e. = standard error; *t* = *t-*value.

fixed effects	*β* coefficient	s.e.	d.f.	*t*	*p*
intercept	43.83	3.05	31.77	14.36	*<0.001*
time	0.23	0.12	360.76	1.81	0.071
fin beats	6.38	0.39	399.81	16.24	*<0.001*
position (P2–P1)	−1.24	1.45	393.81	−0.86	0.393
position (P3–P1)	−7.90	1.44	392.55	−5.50	*<0.001*
position (P3–P2)	−6.65	1.43	391.83	−4.67	*<0.001*
random effects	variance	s.d.			
individual (intercept)	35.55	5.96			
residuals	135.98	11.66			

**Figure 4.**
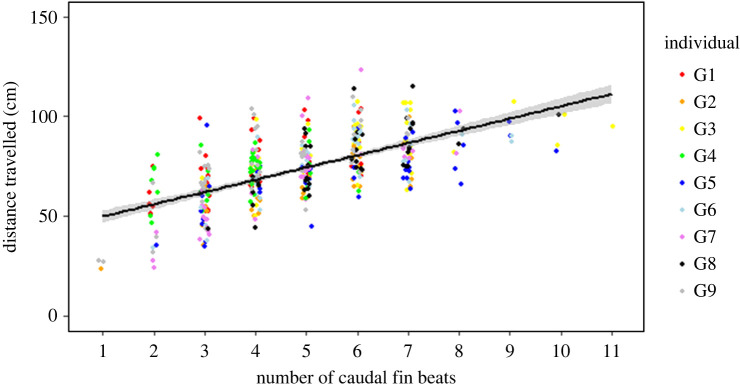
Effect of the number of caudal fin beats on the travelled distance. The black line and grey shading represent the linear regression ± s.e. The positive slope *R* = 62.3% indicates that fin beats is positively correlated to distance travelled. (Online version in colour.)

The number of caudal fins beats significantly and positively correlated to the swimming distance (table 2; figure 4), so that the greater the distance travelled, the more fin beats occurred. The ratios of the coefficient of variation between fin beats number and distance travel are ranging from 1.12 to 1.94. Ratios close to one indicate that the fish were similarly consistent in their distance estimation and their fin beats number across trials. Ratios closer to two indicate that the variation in the number of fin beats produced was twice as large as the variation in the distance estimate (table 3).

**Table 3.** Coefficient of variation of distance travelled and number of fin beats for each individual.

individual	distances travelled CV	number of fin beats CV	CV Ratio
G1	19.51	30.08	1.54
G2	21.91	28.74	1.31
G3	16.42	28.64	1.74
G4	14.57	28.21	1.94
G5	23.16	35.75	1.54
G6	21.34	30.52	1.43
G7	29.94	33.67	1.12
G8	20.04	24.50	1.22
G9	26.35	37.25	1.41

We did not observe a significant effect of travel time on goldfish distance travelled (table 2). Moreover, the ratio of coefficient of variation between the travel time and the distance travelled ranged from 1.29 to 3.03 (table 4). On average, the ratio of coefficient of variation was twice as large for travel time than for distance travelled indicating that goldfish were much more consistent in their travel distance estimation than in their travel time. We did not find any effect of the test order on the travel time of individuals (*p* = 0.614; see electronic supplementary material, table A3 for details).

**Table 4.** Coefficient of variation of distance travelled and travel time for each individual.

individual	distances travelled CV	time CV	CV ratio
G1	19.51	37.51	1.92
G2	21.91	31.15	1.42
G3	16.42	31.25	1.90
G4	14.57	22.95	1.57
G5	23.16	58.58	2.53
G6	21.34	49.50	2.32
G7	29.94	43.77	1.46
G8	20.04	25.89	1.29
G9	26.35	79.78	3.03

### (d) The use of background visual information

**Figure 5.**
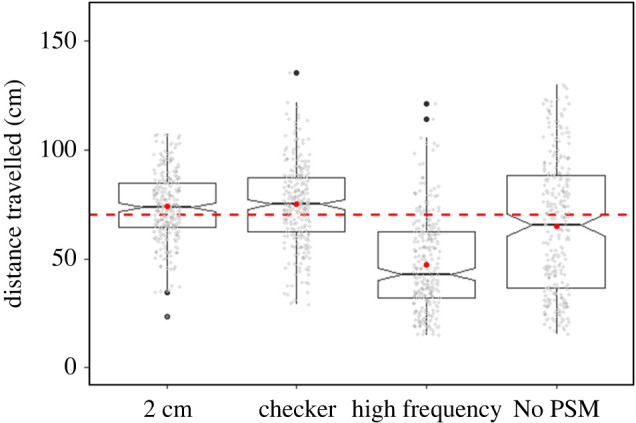
Goldfish estimated distance following visual background alteration. The dashed line represents the 70 cm target distance. Red dots indicate the mean distance estimate for each pattern. Raw data are represented by the grey dots. No PSM = no periodic spatial modulation. *n* = 6 fish, see electronic supplementary material, figure A4 for individual details. (Online version in colour.)

**Figure 6.**
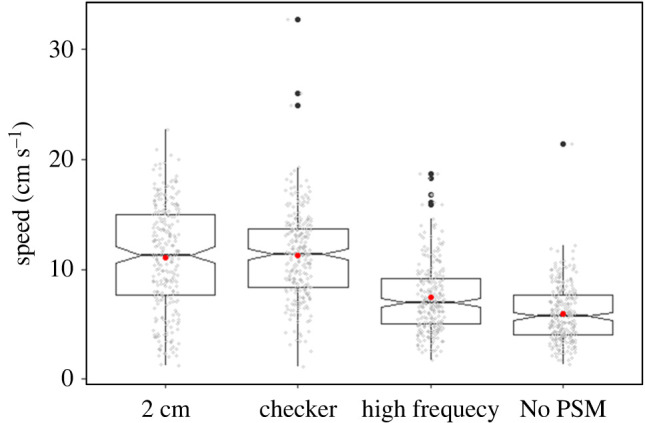
Goldfish swimming speed following alteration of their visual background. Red dots indicate the mean distance estimate for each pattern. Raw data are represented by the grey dots. No PSM = no periodic spatial modulation. *n* = 6 fish. (Online version in colour.)

Six individuals were tested with the four previously described optic flow patterns: 2 cm, checker, high spatial frequency and no periodic spatial modulation. We did not find a significant difference in distance travelled when goldfish were tested with the 2 cm (73.94 ± 15.51 cm) or the checker pattern (74.94 ± 18.79 cm; figure 5; table 5). The fish swam a significantly shorter distance (47.46 ± 21.47 cm) when they were tested with the high spatial frequency pattern than with any other pattern. The distance travelled with the no periodic spatial modulation pattern (65.04 ± 30.55 cm) was significantly shorter than with the 2 cm and checker patterns and significantly longer than with the high spatial frequency pattern. Moreover, the goldfish showed much more variability in their distance estimate with no periodic spatial modulation, with a standard deviation twice as large as for the 2 cm pattern, representing almost half of their travelled distance. Individual travel distance for each pattern is given in the electronic supplementary material, table A1. Details of the distance travelled with the four different patterns for each of the six individual and for the three start positions are presented in the electronic supplementary material, figure A4 and A5, respectively. Goldfish swimming speed was also significantly slower when they were tested with the high spatial frequency (7.44 ± 3.21 cm s^−1^) and no periodic spatial modulation (5.95 ± 2.57 cm s^−1^) patterns, compared to the 2 cm (11.08 ± 4.75 cm s^−1^) and checker patterns (11.28 ± 3.99 cm s^−1^, figure 6, see electronic supplementary material, table A4 for *p*-values and model summary).

**Table 5.** Effect of the background pattern on the travel distance. Results from the linear mixed model with fish ID and position as crossed random intercept. Significant results are in italics. d.f. = degrees of freedom; *p* = *p-*value; s.d. = standard deviation; s.e. = standard error; *t* = *t-*value; PSM = periodic spatial modulation.

fixed effects	*β* coefficient	SE	DF	*z*	*p*
intercept	73.94	4.16	7.69	17.77	*<0.001*
checker—2 cm	1.00	1.79	1069	0.56	0.576
high spatial frequency—2 cm	−26.48	1.79	1069	−14.81	*<0.001*
no PSM - 2 cm	−8.90	1.79	1069	−4.98	*<0.001*
high spatial frequency—checker	−27.48	1.79	1069	−15.36	*<0.001*
no PSM—checker	−9.90	1.79	1069	−5.53	*<0.001*
no PSM—high spatial frequency	17.58	1.79	1069	9.83	*<0.001*
random effects	variance	s.d.			
individual (intercept)	66.89	8.18			
position (intercept)	13.67	3.70			
residuals	431.85	20.78			

## 4. Discussion

Distance is an important metric that underpins spatial cognition, and we suggest that this behaviour can be exploited to study the evolution of spatial cognition from behaviour through to the neural systems that underlie it. In this study, we have found that goldfish are able to accurately reproduce a learned distance and that they can use optic flow information to do so. Goldfish were trained to travel a distance of 70 cm before returning to a start position and continued to do so, during testing, when external cues (experimenter waving) were removed (average swimming distance = 74.0 ± 16.7 cm). Although distance estimation accuracy was impacted by start position, it is unlikely that fish used a fixed landmark cue (external or internal to the experimental tank) for distance estimation because they were able to swim the approximate learned distance at all start positions (20 cm apart) and therefore were turning at different absolute positions depending on the start position. This first result indicates that the spatial metric of distance is encoded by goldfish and that this species provides a robust model system for future examinations of the neural basis of spatial cognition in teleost fish, allowing to link together behaviour with the neural mechanism that drives it.

The ability to estimate distance in fish was recently shown in Picasso triggerfish [27], but there were some interesting differences between that species fish and the goldfish. First, the goldfish were not as accurate as the triggerfish when estimating distance travelled. The triggerfish were trained to swim 80 cm and travelled an average of 80.3 ± 3.7 cm. Multiple factors could explain this difference. First, the triggerfish may encode distance more accurately than goldfish. This difference in ability could simply be species-specific but could also be related to the difference in environmental rearing conditions (see [35] for a review). While the goldfish were reared in captivity, the triggerfish came from a wild population and therefore likely experienced higher variation in environmental stimuli. It has been shown that rearing and enrichment conditions can significantly impact forebrain development and cognitive abilities in salmon [36,37]. In Chinook salmon (*Oncorhynchus tshawytscha*), wild-caught individuals had significantly larger olfactory bulb and telencephalon volumes relative to their body size compared to individuals reared in hatcheries [36]. In Atlantic salmon (*Salmo salar*), individuals reared in enriched environments (e.g. pebbles, rocks and floating structures) were more accurate at locating the exit of a maze compared to individuals reared without [37]. Second, triggerfish might be more accurate in distance estimation because of the ecological relevance of the task. This territorial species requires accurate spatial memory to travel back to their own home after foraging trips. Moreover, knowledge of the position and distance to the nearest shelter would be valuable when encountering predators. Finally, small differences in experimental training and testing could have had an effect. For example, when the triggerfish reached the target distance, it triggered an infrared detector and activated a flashing light training cue. This training set-up was not possible for the goldfish as the detector was not accurate for their given size and propensity to swim at the bottom and out of range of the infrared signal. Instead, the experimenter waved at the goldfish to indicate that the target distance was reached. The millisecond of latency for the experimenter to wave might have given the fish a less accurate idea of the target distance (waving-turning latency information are given in the electronic supplementary material §3). However, the experimenter never waved at the fish during test trials; therefore, any differences in swimming distance between test trials could not result from a difference in waving-turning timing.

The modification of the visual information displayed in the background significantly affected the distance travelled by the goldfish, providing evidence for the use of optic flow as a cue for distance estimation. When the translational optic flow information was removed or substantially reduced (no periodic spatial modulation pattern), distance estimations become inconsistent, with a s.d. that doubled compared to the 2 cm optic flow pattern and represented half of the distance travelled. This demonstrates that optic flow information is crucial in Goldfish distance estimation. When the spatial frequency of the background pattern was increased (high spatial frequency pattern), goldfish overestimated the distance they travelled by 36%, and turned before reaching the target distance. Goldfish demonstrated the same behavioural pattern when presented with the checker pattern (same spatial frequency than the 2 cm pattern) and the 2 cm pattern, indicating that it was the change of spatial frequency and not the change of pattern that altered individual swimming distance. These results suggest that the visual odometer of goldfish is mediated by a movement detection mechanism similar to that underlying optomotor response [38]. The use of optic flow to estimate distance travelled is widespread in other vertebrate and invertebrate species. Humans, ants, wolf-spiders and honeybees use ‘global’ optic flow as a visually guided odometer [22–24,39]. However, they integrate the image motion (i.e. speed of visual background on the retina) and not the structure of the background (i.e. frequency of the optic flow) to estimate distance. In those cases, the change in spatial frequency did not affect individual distance estimation scores. However, changes in either speed or distance to the visual background would affect distance perception. These results combined with our own, indicate that visually based distance estimation is widely spread across taxa but that the visual information extracted (frequency versus angular speed) differs from fish compared to insect or mammals. The similarity of goldfish and triggerfish behaviour in response to changes in optic flow cues (i.e. high spatial frequency pattern = overestimated travel distance, no periodic spatial modulation = reduced accuracy) [28]) suggests that the use of background spatial frequency information, ‘local optic flow’, as an optometer may be widespread among visually oriented teleosts. Particularly given that the two species inhabit different visual environments [40–42]. Notwithstanding the diversity in visual cues, water environments such as ponds, rivers and reefs display a high variety of visual features (e.g. submerged branches, algae, rocks and corals) that are likely to provide enough spatial modulation to allow fish to estimate distance using optic flow. Alternatively, species inhabiting dark (e.g. caves and deep water) or highly turbid environments are likely to exploit alternative sensory mechanism (such as counting fin beats number or gathering information from the lateral line) to estimate distance.

In contrast with the results obtained with triggerfish, goldfish swimming speed was significantly affected by changes in the spatial frequency of the background pattern. In goldfish, the travel distance and travel speed both decreased by 36% and 33%, respectively, when the spatial frequency was doubled. We could wonder if goldfish were using time as a proxy to estimate distance, swimming a shorter distance slower. However, supplementary analyses showed that goldfish swimming time was significantly different for each background pattern tested. If individuals were using time as a proxy to estimate distance, we would expect the travel time to be consistent across the background patterns tested. Instead, we found that individuals swam a significantly longer amount of time with the 2 cm pattern (electronic supplementary material, table A5 for details). Because travel distance and travel speed were both affected by the spatial frequency of the visual background we can assume that the rate of movement of visual contrast might be used in the same way for speed control and for odometry. The visual control of swimming speed and odometry by goldfish could therefore be underpinned by the same visual motion mechanisms. This mechanism could be shared with other species such as the honeybees, who use optic flow cues to control their speed [43]. The use of optic flow information allows honeybees to adjust both their speed and distance travelled when foraging in complex environments, and a similar mechanism may confer similar benefits to fish. Interestingly, for teleost fish speed correlated cells have recently been found in the goldfish lateral pallium when they are freely navigating within a confined space [11]. However, there was no periodic spatial modulation pattern presented in this study so the lateral line was the most likely sensory system to inform individuals about their swimming speed. Our results indicate that the information gathered by both the lateral line and the individual visual system could inform goldfish about their swimming speed. A recent review pointed out that spatial memory in teleost fish could be possibly considered a special case of a wider relational memory system that encodes both the spatial and the temporal dimensions of episodic-like memories and that those encoding takes place in the hippocampal pallium of teleost fish [8].

Along with optic flow information, goldfish could use alternative cues to estimate distance. We explored the role of the start position, the number of fin beats and the travel time in goldfish distance estimation. Goldfish performed significantly better (mean travelled distance closer to the target distance and smaller s.d.) when tested at the third position than at the first or second position. When tested at the third position, the absolute turn position was closer to the end of the experimental tank making it impossible to swim beyond 30 cm after the 70 cm target distance before reaching the end of the experimental tank. When tested at the first start position, they were able to swim 70 cm after the 70 cm target distance. This might be a confounding effect leading to a better accuracy (lower s.d.) when tested at the third position. Moreover, it is possible that individuals used a combination of self-integrated distance measurement and spatial cues to obtain better accuracy in their travelled distance estimate. The closeness to the end of the tunnel could give them supplementary information about their turning point. Studies have shown that multiple fish species (e.g. redtail splitfins, *Xenotoca eiseni*, and goldfish) were able to use the geometry of space (geometrical cues of a rectangle arena) in navigation and decision making [44–46]. In a spontaneous reorientation task (i.e. where no reinforced training was used), redtail splitfins and zebrafish (*Danio rerio*) could also use geometrical information to find the previous position of a conspecific [47,48]. Recent neurophysiological work by Vinepinsky *et al*. [11] has revealed the presence of edge detection neurons in the lateral pallium of goldfish, which is a first indicator that teleost fish are able to encode information about the features of space.

The number of caudal fin beats was significantly correlated to the swimming distance. The ratio of the variation of fin beat number and travelled distance ranged from 1.1 to 1.9. Variation in fin beat number was therefore greater than the variation in travelled distance for each individual. However, for five individual, the ratio was below 1.5. Individuals with a ratio of 1.1 and 1.2 had similar variation in distance estimate and fin beat number. It is thus possible that some individuals used fin beats as an odometer and a proxy to estimate travel distance. Individual differences in navigation strategies have been observed in other fish species. For example, Lee *et al*. [47] found species (redtail splitfins versus zebrafish) and sex difference in the use of landmark in a reorientation task. While both sexes used geometrical cues to orient, the combined use of landmark and geometry was only significant in males. Females only showed the use of geometry without distinction between the two geometric twins. Males using both geometrical and landmark cues were therefore more accurate but only when the goal was nearer to the attractive local landmark (they used the landmark as a beacon). Moreover, redtail splitfins were successful at using both landmark and geometrical cues while zebrafish used mainly geometrical cues. The ratio of variation in fin beat number versus variation in distance travelled was much lower in goldfish than in the triggerfish [27]. Species differences in navigational strategies could explain the fact that triggerfish were not using fin beats to measure distance. This species-specific strategy could be explained by the subcarangiform swimming pattern in goldfish and the oscillatory swimming style of triggerfish. In other species, while the honeybees (*Apis mellifera*) odometer is driven by image motion [49], desert ants (*Cataglyphis)* and fiddler crab (*Uca pugilator*) use step count (pedometer) as an odometer [15,16]. A definitive answer as to whether goldfish use fin beats as odometer requires further investigation.

Goldfish were unlikely to use time as a proxy to estimate distance travelled. We did not find a significant effect of time on the distance travelled and the coefficient of variation in time travelled was two to three times higher than the coefficient of variation in distance estimate for most fish. Video analysis revealed high variation in time spent to perform the experiment within a day or a session without any apparent pattern, further supporting this hypothesis. The trial order did not significantly impact the time spent to perform the task. This result is consistent with triggerfish [27] that also did not use time as a proxy to estimate the travelled distance. Studies with other taxa also reported that time was uncorrelated with travelled distance. For example, honeybees did not use time to estimate flight distance when tested in a tunnel with either headwind or tail wind [24,50]. Moreover, in humans, the variability at reproducing a given distance by walking was found to be lower than the variable to estimate an interval of time. This result did not favour a temporal metric for locomotor distance perception in humans because their precision in distance production was greater than their precision of temporal interval [51]. If individuals used time as a proxy to measure distance travelled, the error produced in the distance estimation task (variation in distance estimate) should be similar to the error in time to perform the task. However, in this experiment, the variability in distance travelled was two times lower than that of travel time. In their natural environment, time may not be an accurate proxy for distance as goldfish are likely to forage and interact with conspecific while travelling from a point A to a point B and therefore high temporal variability during their travel is likely to occur. Flow within aquatic systems could also disrupt this estimate.

In conclusion, we have shown that goldfish use the spatial frequency of the visual background for odometry and speed control. The two processes might be underpinned by a similar motion detection mechanism and are likely to share a similar neural pathway. This study identifies the goldfish as a robust model species to examine the neural basis of spatial cognition in teleost fish and advances the use of this animal system to understand the evolution of spatial mechanisms (neural and behavioural).

## Data Availability

Data and codes used are provided in the Dryad Digital Repository: https://doi.org/10.5061/dryad.0k6djhb2s [52]. The data are provided in the electronic supplementary material [53].
